# Abnormal resting-state functional connectivity of hippocampal subfields in patients with major depressive disorder

**DOI:** 10.1186/s12888-020-02490-7

**Published:** 2020-02-17

**Authors:** Zi Yu Hao, Yuan Zhong, Zi Juan Ma, Hua Zhen Xu, Jing Ya Kong, Zhou Wu, Yun Wu, Jian Li, Xin Lu, Ning Zhang, Chun Wang

**Affiliations:** 1 Nanjing Brain Hospital Affiliated to Nanjing Medical University, Nanjing, 210029 Jiangsu China; 2 School of Psychology, Nanjing Normal University, Nanjing, 210097 Jiangsu China; 3 Jiangsu Key Laboratory of Mental Health and Cognitive Science, Nanjing Normal University, Nanjing, 210097 People’s Republic of China; 4 Functional Brain Imaging Institute of Nanjing Medical University, Nanjing, 210029 Jiangsu China; 5 Cognitive Behavioral Therapy Institute of Nanjing Medical University, Nanjing, 210029 Jiangsu China

**Keywords:** Major depressive disorder, Hippocampal subfields, Cornu ammonis, Dentate gyrus, Subiculum, Resting-state functional connectivity

## Abstract

**Background:**

Many studies have found that the hippocampus plays a very important role in major depressive disorder (MDD). The hippocampus can be divided into three subfields: the cornu ammonis (CA), dentate gyrus (DG) and subiculum. Each subfield of the hippocampus has a unique function and are differentially associated with the pathological mechanisms of MDD. However, no research exists to describe the resting state functional connectivity of each hippocampal subfield in MDD.

**Methods:**

Fifty-five patients with MDD and 25 healthy controls (HCs) matched for gender, age and years of education were obtained. A seed-based method that imposed a template on the whole brain was used to assess the resting-state functional connectivity (rsFC) of each hippocampal subfield.

**Results:**

Patients with MDD demonstrated increased connectivity in the left premotor cortex (PMC) and reduced connectivity in the right insula with the CA seed region. Increased connectivity was reported in the left orbitofrontal cortex (OFC) and left ventrolateral prefrontal cortex (vlPFC) with the DG seed region. The subiculum seed region revealed increased connectivity with the left premotor cortex (PMC), the right middle frontal gyrus (MFG), the left ventrolateral prefrontal cortex (vlPFC) and reduced connectivity with the right insula. ROC curves confirmed that the differences between groups were statistically significant.

**Conclusion:**

The results suggest that the CA, DG and subiculum have significant involvement with MDD. Specifically, the abnormal functional connectivity of the CA may be related to bias of coding and integration of information in patients with MDD. The abnormal functional connectivity of the DG may be related to the impairment of working memory in patients with MDD, and the abnormal functional connectivity of the subiculum may be related to cognitive impairment and negative emotions in patients with MDD.

## Background

Major depressive disorder (MDD) is a psychiatric illness that seriously affects society and life [34, 43]. MDD affects 350 million people worldwide each year (WHO, 2017), and its lifetime prevalence can reach 3.4% in China [76]. The main symptoms of MDD include persistent negative effects, loss of power, inattention and increased guilt and appetite, which are associated with abnormal brain function and structure [5]. Furthermore, the WHO predicts MDD will become the world’s second-largest disease by 2020 [46]. However, the pathophysiology of the disease is still unclear and the recurrence rate is very high.

The hippocampus is a core component of the limbic-cortical dysregulation model of MDD, which is involved in the foundation of MDD neurobiology and plays a very important role in memory and cognitive function [25, 41, 51]. Moreover, the hippocampus also plays an important role in the regulation of stress and emotion [18]. Therefore, the memory deficits and depression experienced by patients with MDD may originate with the hippocampus [40, 58]. Unsurprisingly, many studies have found abnormal activation of the hippocampus in patients with MDD [32, 44]. These findings indicate that the hippocampus may be involved in the neurobiological basis of MDD.

Recently, it was discovered that the hippocampus can be divided into three different subfields for research. This is because the different subfields of the hippocampus have distinct functions [65]. The three subfields are: the cornu ammonis (CA), dentate gyrus (DG) and subiculum [17] [1]. The CA is related to learning and memory functions in humans and other mammals, and mainly participates in short-term image contact, image formation and fear memory formation. In addition, the CA plays an important role in medium-term and short-term spatial memory [28]. The DG, on the other hand, is the receptacle for incoming spatial information to the hippocampus. At the same time, it also processes and encodes spatial information, which plays an important role in spatial learning and memory [33]. Meanwhile, the subiculum is the main component of hippocampal information output, transmitting information processed from the DG to the corresponding neuroendocrine system and is also the effector of the baroreceptor reflex [47].

The hippocampus is composed of several subfields that are differentially associated with MDD [72]. However, most studies of MDD in hippocampal subfields focus on volume. Many studies have reported smaller hippocampal subfield volumes compared to healthy controls [9, 10, 26] however, smaller hippocampal subfield volumes in patients with MDD is not a universal phenomenon, so it is controversial to use hippocampal subfield volume as a biomarker of depression [3, 16, 59]. Therefore, it is not enough to study the changes of hippocampal subfield volume in patients with MDD. We should also explore the differences in functional connectivity of hippocampal subfields between groups.

fMRI research has increased dramatically in recent years, especially in the field of resting-state functional connectivity (rsFC). rsFC represents the temporal coherence of the blood-oxygen-level-dependent (BOLD) signal within or between brain regions or networks during rest, and subsequently helps to reveal the neurobiological basis of MDD [45, 62]. Seeing that many studies have found that some symptoms of MDD are related to abnormal rsFC [30, 69], this article uses the method of resting-state fMRI to explore rsFC of hippocampal subfields.

The aim of this study was to extend our understanding of the role of hippocampal subfields in MDD by examining rsFC of each hippocampal subfield with the whole brain along with differences in rsFC of hippocampal subfields between patients with MDD and HCs. We hypothesized that rsFC of each hippocampal subfield would differ between patients with MDD and HCs and that rsFC would further elucidate the differences between the CA, DG and subiculum.

## Method

### Participants

Patients with MDD were recruited from the Department of Medical Psychology of Nanjing Brain Hospital, affiliated with Nanjing Medical University. HCs were recruited from society through advertising and matched with MDD patients in terms of age, gender and education.

Inclusion criteria for patients with MDD included: (1) conformation to the DSM-IV diagnostic criteria of MDD, (2) 20–50 years old, (3) in their first onset, (4) Scores ≥18 on the 24-item version of the Hamilton Rating Scale for Depression (HAMD, Hamilton M, 1960), (5) right-handed, (6) voluntary participation and signed informed consent. Exclusion criteria for patients included: (1) patients with other psychotic disorders, severe physical illness or infectious diseases, (2) substance abuse, (3) current pregnancy, (4) MRI contraindications, (5) patients who received systemic drug therapy, psychotherapy or electroconvulsive therapy within 6 months prior to enrollment.

HCs met the following criteria: (1) 20–50 years old, (2) right handed, (3) voluntary participation, and signed informed consent. The exclusion criteria for HCs included: (1) people with nervous system disease, mental illness or serious physical illness, (2) personal history/family history of psychiatric disorders or psychiatric illness, (3) have taken psychotropic drugs or had psychological counseling within the past 3 months, (4) current pregnancy, (5) MRI contraindications.

### fMRI data acquisition and processing

All rs-fMRI data were acquired on a Siemens Verio MRI 3.0 Tesla scanner. Before scanning, foam pads and earplugs were used to reduce head movement and noise. All subjects were instructed to stay awake and close their eyes during the scan. In order to reduce data errors, subjects whose heads exceeded 3° of motion were rejected. fMRI scanner parameters: gradient-echo and echo-planar-imaging, T1-weighted structure image, TR = 1900 ms, TE = 2.48 ms, flip angle = 90°, FOV = 250 mm, matrix = 256 × 256, 176 slices, slices thickness/gap = 1.0 mm/0.5 mm. T2-weighted functional image: TR = 3000 ms, TE = 40 ms, flip angle = 90°, FOV = 240 mm, matrix = 64 × 64, 32 slices, slice thickness/gap = 4 mm/4 mm, total time = 8min6s.

Rs-fMRI data were preprocessed using Data Processing Assistant of Resting State fMRI (DPARSF) within the MATLAB toolbox, which is based on SPM (Statistical Parametric Mapping) and REST (Resting-State fMRI Data Analysis Toolkit) (http://restfmri.net/forum/). Data preprocessing included: realignment and head motion correction (head motion or rotation greater than 3° would be excluded), spatial normalization (the functional images were spatially normalized to MNI (Montreal Neurological Institute) template and resampled to 3*3*3 mm3) and smoothing (full width at half maximum, FWHM = 6 mm*6 mm*6 mm). Then, detrending and filtering (0.01~0.08) were used to remove high-frequency physiological noise and low-frequency drift. We also regressed out nuisance covariates including 6 head motion parameters, global mean signal, cerebrospinal fluid signal and white matter signal. The group differences of head motion were assessed with the two sample t-test according to the following formula:

Head Motion/Rotation== $\frac{1}{L-1}{\sum}_{i=2}^L\sqrt{{\left|{x}_i-{x}_{i-1}\right|}^2+{\left|{y}_i-{y}_{i-1}\right|}^2+{\left|{z}_i-{z}_{i-1}\right|}^2}$ [78]. The results indicated that there was no significant distinction between the two groups (two sample t-test, *t* = −1.3101, *p* = 0.1943 for translation, and *t* = −1.3132, *p* = 0.1933 for rotational).

### Hippocampal subfields definition

**Fig. 1:**
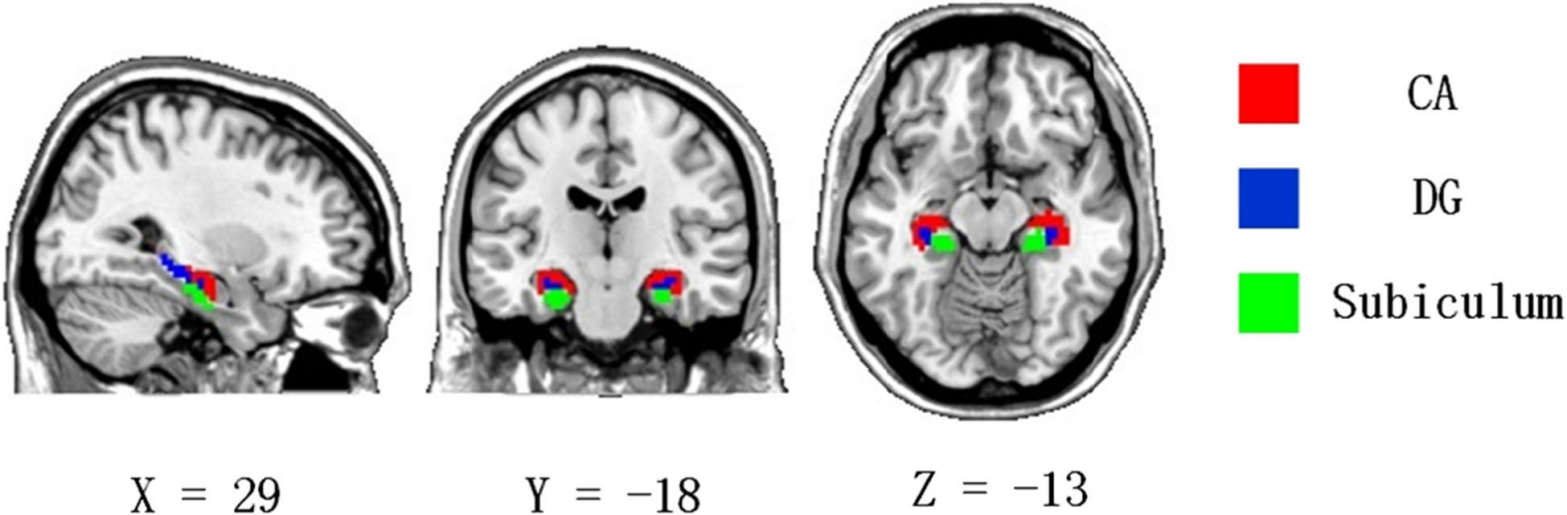
Hippocampal subfields. CA, cornu ammonis (shown in red); DG, dentate gyrus (shown in blue); subiculum (shown in green)

The hippocampal subfield template we used divided the hippocampus into three subfields: CA, DG and subiculum (Fig. 1). The hippocampal subfields were divided by probabilistic maps that had organizational structure boundaries. Then, they were resliced according to the spatial voxel size of 3 mm × 3 mm × 3 mm and selected so that at least 50% of the voxels fell into the seed area to make the final seed area. Each voxel belonged to only one seed area. Then the time course of the seed area was extracted after averaging the BOLD signals of the bilateral seed areas, respectively, for the following functional connectivity analysis.

### Data analysis

There were no significant differences in age, years of education or gender between groups at a significance level of *P* < 0.05. In contrast, there were significant differences in HAMD score between the two groups (Table 1). Analyses were based on the Statistical Package for the Social Sciences25 (SPSS25) (https://www.ibm.com/analytics/spss-statistics-software**).**

**Table 1:** Comparison of demographic and clinical variables among MDD and HCs

Variable	MDD (*n* = 55)	HC (*n* = 25)	t/χ2	p
Gender (males/females)	20/35	13/12	1.315	0.193
Age	34.80 ± 9.04	38.24 ± 10.13	−1.561	0.123
Education	14.12 ± 2.03	14.32 ± 2.75	−0.329	0.744
HAMD	25.00 ± 5.16	2.72 ± 1.46	29.548	0.000

*MDD* major depressive disorder, *HCs* healthy controls, *HAMD* Hamilton Depression Scale

After processing, individual- subject level voxel-wise analyses were built in the REST toolbox (Song et al., 2011) by using Fisher’s r-to-Z transform to convert the data to Z-scores. In order to identify the differences in rsFC of each hippocampal subfield with the whole brain and the differences in rsFC of hippocampal subfields between patients with MDD and HCs, rsFC analysis was performed using the second-level model in SPM8.

Finally, the ROC curve, which can discriminate patients with major depressive disorder from healthy controls, was drawn by using Z scores of hippocampal subfield rsFC with between-group differences. The ROC curve was done in SPSS25, with a larger area under the curve (AUC) providing more accurate results. The Z scores of rsFC that were extracted from each voxel in significant clusters were also used to test the correlation between HAMD scores and functional connections through Pearson linear partial correlation at 95% confidence level in SPSS25.

## Results

### Demographics

Demographic and clinical data were collected from participants upon recruitment. Patients with MDD and HCs were compared in terms of gender, age, HAMD scores and years of education. While there were no significant differences in gender, age or years of education, there was a significant difference in HAMD scores; HAMD scores in patients with MDD were significantly higher than HCs (Table 1).

### RsFC of hippocampal subfields

**Fig. 2:**
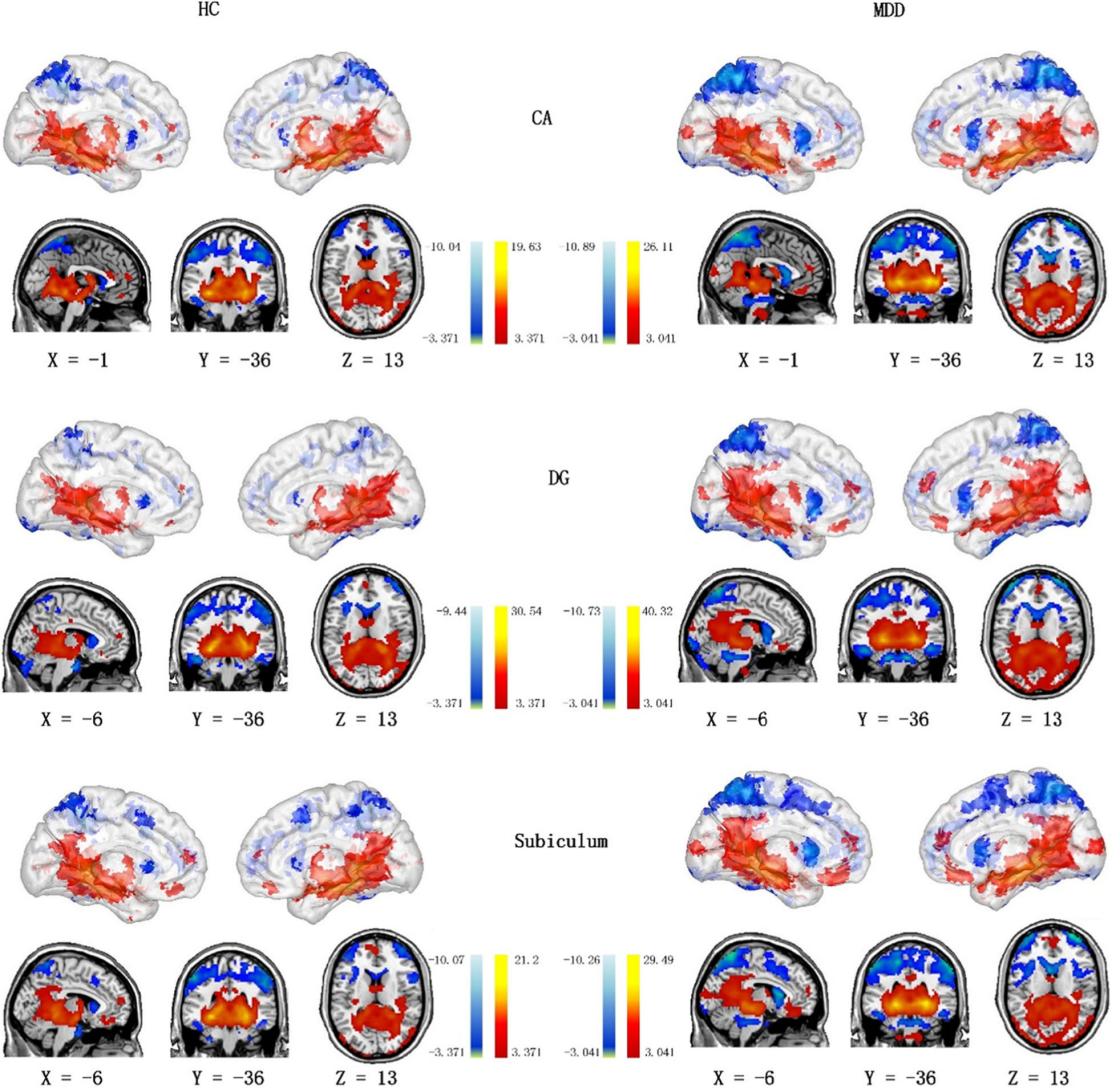
Functional connectivity maps of each hippocampal subfield in MDD and HCs. The results were corrected using the FDR method (threshold of P < 0.01). Color-bar represents t-values

Based on results from the one-sample t test, we observed positive functional connectivity between the hippocampal subfields (CA, DG and subiculum) and a wide range of brain regions including the hippocampus, lingual gyrus, inferior temporal gyrus, amygdala, middle occipital gyrus, orbitofrontal cortex (OFC) and medial prefrontal cortex (mPFC). Negative functional connectivity was noted between the hippocampal subfields and the insula, posterior parietal cortex (PPC) and dorsolateral prefrontal cortex (dlPFC) (Fig. 2 and Table 2).

**Table 2:** Resting-state functional connectivity of each hippocampal subfield

Seed	Brain region	BA	voxel	t
CA	mPFC	10	35	5.51
	Lingual Gyrus	29	256	19.63
	Temporal_Inf_L	21	115	19.63
	Amygdala	34	75	19.63
	Hippocampus	35	75	19.63
	Occipital_Mid_R	19	122	19.63
	ACC	24	34	5.80
	dlPFC	10	306	−6.09
	Insula	13	37	−9.33
	PPC	40	233	−9.33
DG	mPFC	11	37	4.62
	Lingual Gyrus	29	328	30.53
	Temporal_Mid_R	21	280	30.53
	Middle Occipital Gyrus	19	172	30.53
	Amygdala	34	76	30.53
	Hippocampus	35	75	30.53
	Left Cerebellum		220	−7.92
	dlPFC	10	148	−7.51
	Insula	13	30	−5.85
	MFG	40	474	−8.35
	PPC	40	281	−8.35
Subiculum	OFC	11	21	6.04
	mPFC	10	50	5.45
	Middle Occipital Gyrus	19	211	21.20
	Lingual Gyrus	29	204	21.20
	Temporal_Inf_L	21	111	21.20
	Amygdala	34	77	21.20
	Hippocampus	35	75	21.20
	dlPFC	10	181	−8.57
	MFC	9	85	−8.57
	PMC	6	73	−8.57
	Insula	13	62	−8.57
	PPC	40	334	−10.07

*mPFC* medial prefrontal cortex, *ACC* anterior cingulate cortex, *dlPFC* dorsolateral prefrontal cortex, *PPC* posterior parietal cortex, *MFG* medial frontal gyrus, *OFC* orbitofrontal cortex, *MFC* medial frontal cortex, *PMC* premotor cortex

Results from the one-sample t test also revealed that there were differences in the rsFC maps of the CA, DG and subiculum. For instance, positive functional connectivity was exhibited by the anterior cingulate cortex (ACC) with the CA seed region. In addition, negative functional connectivity was reported between the DG seed and the left cerebellum. Finally, negative functional connectivity was demonstrated between the subiculum seed and the medial frontal cortex (MFC) and right premotor cortex (PMC) (Fig. 2). The results were corrected using the FDR method (threshold of *P* < 0.01).

### RsFC alterations between patients with MDD and HCs

**Fig. 3:**
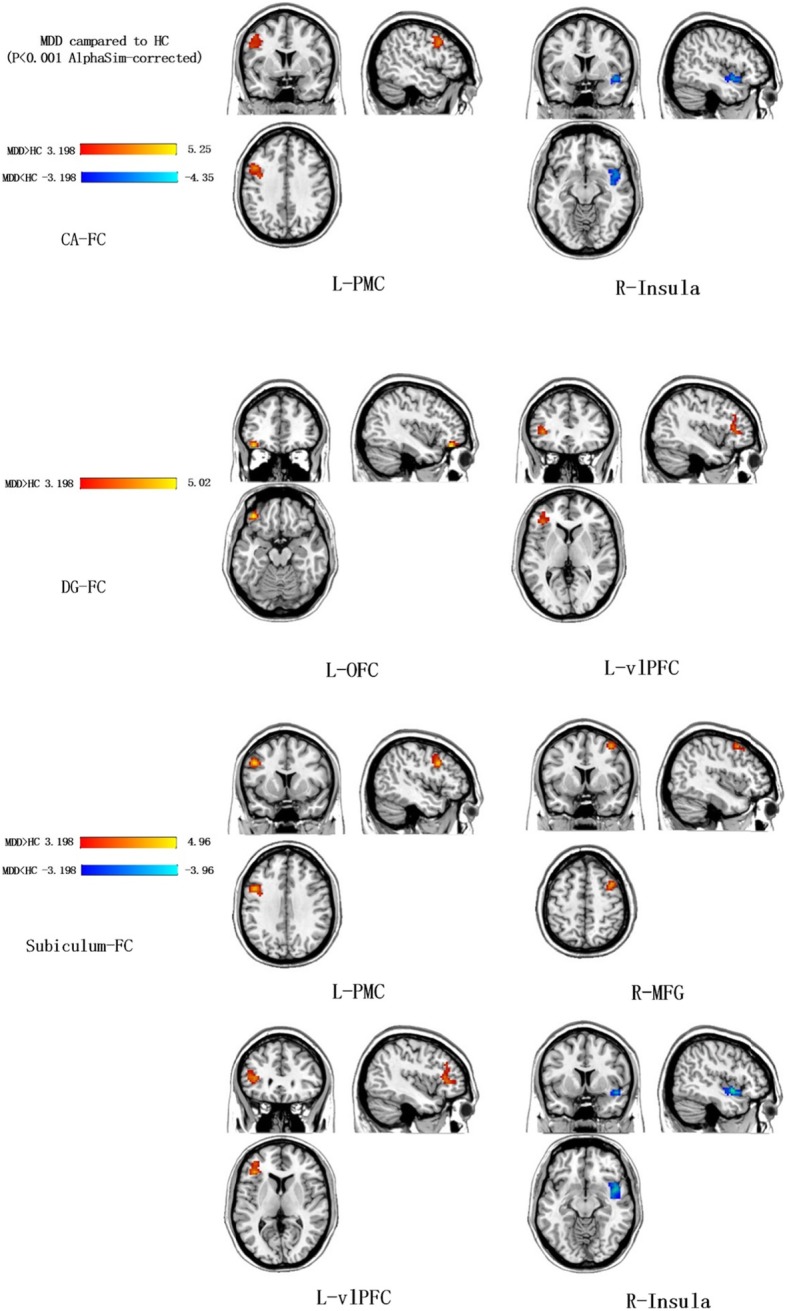
The rsFC alterations of each hippocampal subfield between MDD and HCs, as determined using a two-sample t-test. Warm colors show increased resting-state functional connectivity and cool colors show decreased resting-state functional connectivity with each hippocampal subfield compared to HC. The color bar represents t-values. MDD: major depressive disorder; HC: healthy control; L-PMC: the left premotor cortex; R-insula: the right insula; L-OFC: the left orbitofrontal cortex; L-vlPFC: the left ventrolateral prefrontal cortex; R-MFG: the right middle frontal gyrus

Compared to HCs, the CA seed region revealed increased functional connectivity with the left PMC in MDD. On the other hand, reduced functional connectivity between the CA and the right insula was also shown. Furthermore, increased functional connectivity was reported between the DG seed and the left OFC and left vlPFC. With the subiculum seed region, increased functional connectivity was revealed between the left PMC, the right MFG and the left vlPFC. In addition, decreased functional connectivity was shown between the subiculum and the right insula (Fig. 3 and Table 3). Results were corrected using the AlphaSim method (threshold of *P* < 0.001, cluster: *p* < 0.05).

**Table 3:** Resting-state functional connectivity of each hippocampal subfield between MDD and HCs

Seed	Brain region	BA	Cluster size	t	Peak MNI coordinates X, Y, Z
CA	L-PMC	9	23	3,87	45, 6,-9
	R-Insula	38	20	−3.63	−45, 12, 36
DG	L-OFC	11	18	5.02	−39, −39, 18
	L-vlPFC		15	3.80	−36, 33, 3
Subiculum	L-PMC	9	35	4.64	−48, 12, 36
	R-MFG	6	17	4.07	39, 9, 54
	L-vlPFC		14	4.08	−39, 33, 6
	R-Insula	38	27	−3.84	45, 3, −9

*MDD* major depressive disorder, *HCs* healthy controls, *L-PMC* the left premotor cortex, *R-insula* the right insula, *L-OFC* the left orbitofrontal cortex, *L-vlPFC* the left ventrolateral prefrontal cortex, *R-MFG* the right middle frontal gyrus

**Fig. 4:**
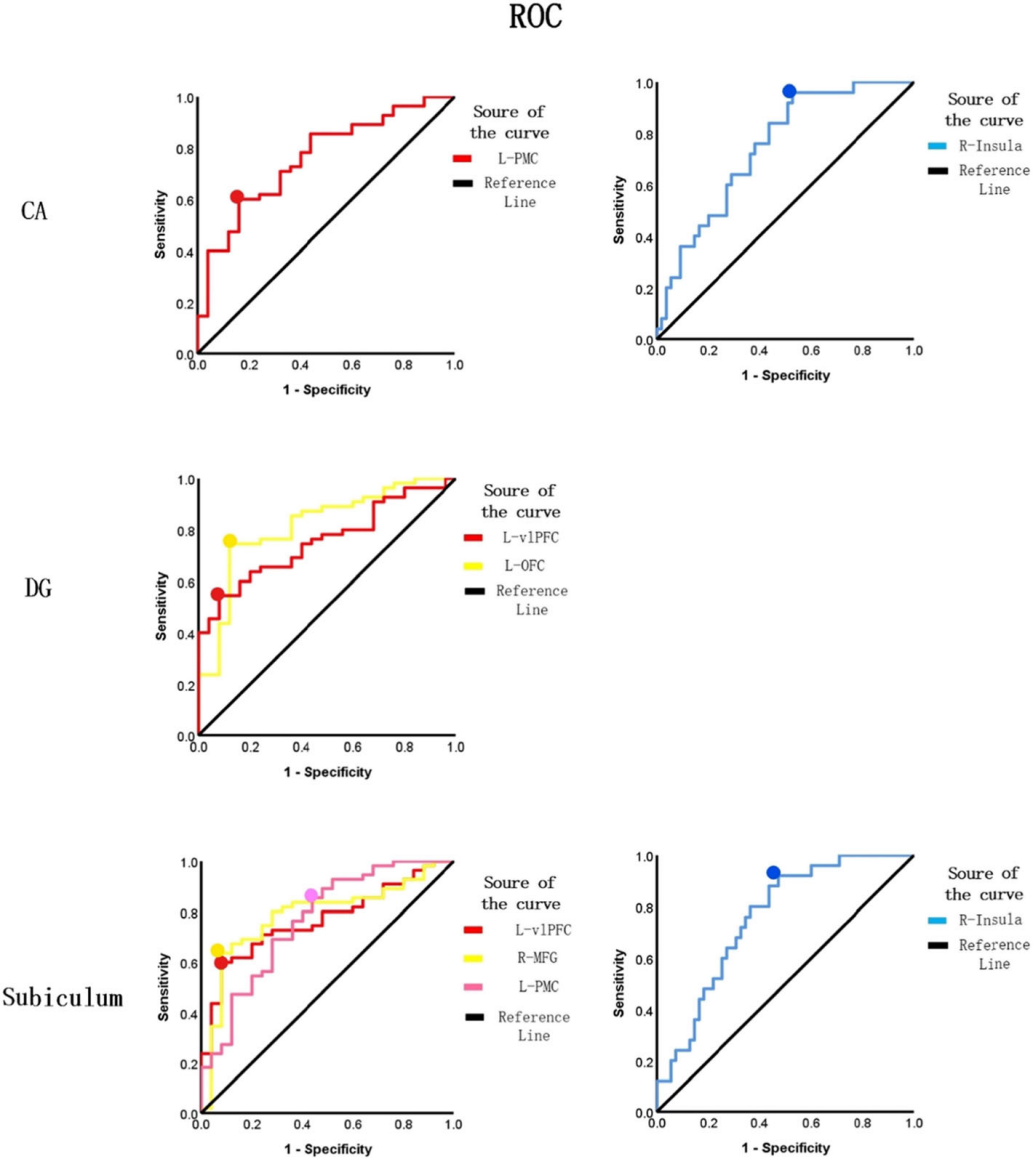
Receiver operating characteristic (ROC) curve discriminated MDD patients from HCs by evaluating Z scores of rsFC with significant between-group differences; enhanced accuracy was noted with a larger area under the curve (AUC). With the CA seed region, L-PMC: the left premotor cortex (AUC:0.76; cutoff: sensitivity:0.60; specificity:0.84); R-insula: the right insula (AUC:0.75; cutoff: sensitivity:0.96; specificity:0.48). With the DG seed region, L-vlPFC: the left ventrolateral prefrontal cortex (AUC:0.76; cutoff: sensitivity:0.55; specificity:0.92); L-OFC: the left orbitofrontal cortex (AUC:0.82; cutoff: sensitivity:0.75; specificity:0.88). With the subiculum seed region, L-vlPFC: the left ventrolateral prefrontal cortex (AUC:0.77; cutoff: sensitivity:0.40; specificity:0.92); R-MFG: the right middle gyrus (AUC:0.77; cutoff: sensitivity:0.64; specificity:0.92); L-PMC: the left premotor cortex (AUC:0.79; cutoff: sensitivity:0.86; specificity:0.56); R-insula: the right insula (AUC:0.76; cutoff: sensitivity:0.92; specificity:0.54)

ROC curves can effectively distinguish between patients with MDD and HCs by measuring area under the curve (AUC), with larger areas providing greater accuracy. In this study, the AUC of the ROC curves of the left PMC and the right insula were 0.76 and 0.75, respectively, when the CA was the seed region. The AUC of the ROC curves of the left OFC and the left vlPFC were 0.82 and 0.76, repectively, when the DG was the seed region. The AUC of the ROC curves of the left PMC, the MFG, the left vlPFC and the right insula were 0.79, 0.77, 0.77 and 0.76, repectively, when the subiculum was the seed region. In addition, the cutoff point for rsFC altertions in hippocampal subfields was more than 1.32 and the cutoff point for the left OFC with the DG seed region was as high as 1.63, which means that the functional circuits of hippocampal subfields have potential to aid in clinical diagnosis (Fig. 4).

## Discussion

This study was designed to assess differences between patients with MDD and HCs in functional circuitry of each hippocampal subfield, the CA, DG and subiculum, based on whole-brain rsFC. This article demonstrated that patients with MDD and healthy controls differed in the rsFC of each hippocampal subfield. Specifically, patients with MDD primarily displayed alterations between hippocampal subregions and the PMC, OFC, vlPFC, MFG and insula.

In MDD patients and HCs, our findings of positive functional connectivity between hippocampal subfields and the hippocampus, lingual gyrus, inferior temporal gyrus, amygdala, middle occipital gyrus, OFC and mPFC are consistent with previous studies [6, 13, 35, 52, 53, 74]. Both the hippocampus and the amygdala are part of the limbic system, thus they are involved in emotional and social processes, along with the temporal gyrus [19]. Although, compared to those brain regions, the mPFC has a stronger ability to regulate emotions and cognition as part of the default mode network (DMN) [66]. Moreover, the temporal gyrus can be combined with the occipital gyrus, OFC and insula to act together on mood regulation and emotional processes [13, 19]. Finally, the lingual gyrus is mainly related to verbal memory and facial emotion recognition [36]. Also consistent with previous studies are our findings of negative functional connectivity between the three hippocampal subfields (CA, DG and subiculum) and the insula, PPC and dlPFC (X. H [11, 45].). The PCC belongs to the posterior DMN, which mainly participates in the process of consciousness and memory through its relationship with the hippocampus [2, 38]. The vlPFC is part of the central executive network (CEN), which plays a vital role in cognitive tasks with a variety of attentional requirements [39]. All of these findings demonstrate that the CA, DG, and subiculum are involved in emotion and cognitive regulation, suggesting that disruption of those functional circuits affects cognition, memory and emotion.

Importantly, there were some differences between the rsFC maps of the CA, DG and subiculum. For instance, the CA demonstrated positive functional connectivity with the ACC. The ACC plays a very important role in working memory [57], which is similar to the function of the CA. The DG displayed negative functional connectivity with the left cerebellum, which has recently been discovered to be associated with visual memory and has the ability to encode visuospatial information [7]. Finally, negative functional connectivity was shown between the subiculum and the MFC and right PMC. The MFC is a region involved in action and motor generation [14]. Similarly, the PMC also has the ability to generate motor plans. Therefore, these differences between the rsFC maps of the CA, DG and subiculum must be caused by the unique function of each hippocampal subfield.

Selecting the CA and subiculum as seed regions revealed increased functional connectivity of the left PMC in patients with MDD compared to HCs. The PMC is generally understood to convert visuospatial information into a motor plan specific to the position and shape of the object [22, 31, 42]. As mentioned above, the CA is mainly involved in the formation of image and spatial memory, similar to the role of the PMC [28]. This helps to explain the increased functional connectivity we observed between the CA and PMC. Moreover, the PMC can also generate avoidance motivation according to expected danger [15], which may be related to the avoidance behavior displayed by patients with MDD. The CA and subiculum seed regions also revealed decreased rsFC in the right insula. The insula is a core brain area that is primarily responsible for integrating cognitive and emotional information [60]. In addition, the dysfunction of insular has been found in psychosis spectrum disorders, especially in the first-episode depression patients. Moreover, the insular cortex is related to the cognitive-affective function [64]. Therefore, the dysfunction of insular cortex will destroy the cognitive and emotional function of depression patients. The insula is also part of the hate circuit, which exhibits reduced activation in response to both positive and negative emotional stimuli, which is also consistent with the results of our study [63]. In addition, the insula is considered a biomarker of MDD, and many studies have found that the functional and structural abnormalities presented by the insula are related to MDD [23, 77]. Therefore, the abnormal connectivity between the CA and the subiculum with the PMC and insula may be related to the symptoms of MDD, providing evidence for the pathogenesis of MDD. Both of these findings are consistent with previous research [4, 21, 45, 49, 68, 70, 75].

The DG seed region revealed increased functional connectivity with the left OFC. The similar functions of the hippocampus and OFC may explain this [71]; for example, since both the hippocampus and the OFC can predict what is going to happen based on the current situation [8, 55], the abnormal connectivity between the DG and OFC may prompt MDD patients to worry excessively about the future. Also, because the OFC is on the receiving end of hippocampal input while the DG is the on receiving end of spatial information into the hippocampus, this relationship further serves to explain why functional connectivity between the DG and OFC is heightened [29, 33]. In addition, many anatomical studies have found that OFC has abnormal gray matter volume. Compared with healthy people, OFC volume of depression patients is smaller. The study also found that Subjects with OFC dysfunction showed personality changes, including behavioral inhibition, emotional instability and reduced motivation [79]. Therefore, it can be concluded that the abnormal connection between DG and OFC may be the main cause of depression patients’ low motivation.

When the DG and subiculum were used as seed regions, increased functional connectivity was shown in the left vlPFC. It comes as no surprise then, that many studies have found that the vlPFC plays an important role in working memory and other cognitive functions [50, 54, 67]. In particular, the left VLPFC is involved in the assessment of emotion and conscious impulse control. So, impaired function of vlPFC can lead to abnormal emotional assessment and excessive self inhibition in patients with depression [20]. And, the vlPFC is responsible for producing a negative emotional experience [37], so excessive activation of the vlPFC can produce unpleasant emotions. Therefore, this abnormal connectivity may give rise to memory loss and negative emotions in patients with MDD.

Finally, with the subiculum seed region, increased functional connectivity with the right MFG was found and this finding is consistent with previous research [56]. The right MFG plays a very important role in attention as the point of convergence between the dorsolateral prefrontal cortex and the ventrolateral prefrontal cortex [27]. In addition, the MFG is also involved in regulating emotion/cognition and contingency awareness [12, 48]. At the same time, the research shows that MFG, as a part of DLPFC, also has abnormal gray matter volume, and the dysfunction of this cortex may result in damage in retrieval of long-distance memory, management of external stimulation, appropriate change of behavior and psychological flexibility [79]. Therefore, the abnormal connectivity between the subiculum and the right MFG may be related to the lack of concentration, cognitive dysfunction and excessive worry and tension experienced by patients with MDD. What’s more, the subiculum and MFG have similar functions, further explaining the increase in functional connectivity.

All of the above findings prove that the CA, DG and subiculum have unique functions and connections with MDD. Moreover, the abnormal connectivity between each hippocampal subfield with other brain regions causes a series of symptoms such as avoidance behavior in life, low self-evaluation, excessive worry about the future, memory loss, tension and bad mood in patients with MDD. More importantly, through the ROC curve, we are more convinced that each hippocampal subfield plays a very important role in the neurobiological basis of MDD. Therefore, the abnormal connectivity between hippocampal subfields and other brain regions may be used as a biomarker for MDD.

This article has some limitations. First, the CA can be divided into CA1-CA4, but since the 3 T MRI scanners we use are lower in resolution, we cannot accurately distinguish the difference in functional connectivity between CA1-CA4. It is hoped that the 7 T MRI can be used in future studies to distinguish the differences between CA1-CA4. Second, the samples we used included only Chinese subjects, so we anticipate that future research can be combined with data from the brainnetome program. Third, this study did not explore the gender differences of functional connections in the hippocampus, so we should pay more attention to gender differences in the future research. Finally, our findings did not correlate with HAMD. It is possible that because most health controls entered the closed environment of the MRI for the first time, we did provoke their frightened and restless instead of MDD patients, thus resulting is an insignificant relationship between the HAMA score with the rsFC of abnormal brain areas in patients with MDD. Therefore, in future research, we should pay more attention to providing emotional guidance to the participants, so that all participants can maintain a peaceful mood, which will make the research results more accurate.

## Conclusion

In conclusion, this study was designed to investigate rsFC of each hippocampal subfield: the CA, DG and subiculum, with the whole brain as well as their rsFC alterations between patients with MDD and HCs. The findings demonstrate that there were rsFC differences between the CA, DG and subiculum and they each have significant relationships with MDD. Thus, this paper emphasizes the importance and contribution of each hippocampal subfield to MDD, as they may be involved in its pathogenesis and can be used as a biomarker to support the clinical diagnosis, treatment and future research of MDD.

## Data Availability

The datasets resting state fMRI scans used and analyzed during the current study are available from the corresponding author on reasonable request.

## Abbreviations

ACC: anterior cingulate cortex
AUC: Areas under curves
CA: Cornu ammonis
DG: Dentate gyrus
dlPFC: Dorsolateral prefrontal cortex
DSM-IV: Diagnostic and statistical manual of mental disorders
EPI: Echo planar imaging
FA: Flip angle
FOV: Field of view
FWHM: Full width at half maximum
HAMA: Hamilton anxiety rating scale
HCs: Health controls
MDD: Major depressive disorder
MFC: Medial frontal cortex
MFG: Medial frontal gyrus
MNI: Montreal Neurological Institute
mPFC: Medial prefrontal cortex
OFC: Orbitofrontal cortex
PMC: Premotor cortex
PPC: Posterior parietal cortex
ROC: Receiver operating characteristic
SPSS25: Statistical package for the social sciences25
TE: Echo time
TR: Repetition time
vlPFC: Ventrolateral prefrontal cortex
